# Simulation model for cost estimation of integrated care concepts of heart failure patients

**DOI:** 10.1186/2191-1991-3-26

**Published:** 2013-11-12

**Authors:** Joerg Schroettner, Alexander Lassnig

**Affiliations:** 1 Institute of Health Care Engineering, Graz University of Technology, Kopernikusgasse 24/I, Graz 8010, Austria

**Keywords:** Decision-analytic model, Discrete event simulation, Economic evaluation, Conventional care, Telemonitoring, Cost estimation, Cost-effectiveness analyses

## Abstract

**Background:**

As a direct result of the population growing older the total number of chronic illnesses increases. The future expenditure for care of chronically ill patients is an ever-present challenge for the health care system. New solutions based on integrated care or the inclusion of telemedical systems in the treatment procedure can be essential for reducing the future financial burden. Therefore a detailed economic model was developed, which enables the comparison of health and cost outcomes for conventional medical care and different integrated care concepts in heart failure treatment.

**Methods:**

F0r modelling, the discrete event technique was used. The model takes outpatient care as well as inpatient care into account to estimate the total occurring costs. It enables the treatment of patients by a physician, a specialist or a clinical ambulance for the simulation of the outpatient care. For inpatient care the model considers the total-costs of the hospitalization and rate of re-admission and furthermore the costs which occur because of special medical treatments or necessary stay at intensive care units. To rate the severity of symptoms patients can be classified using NYHA groups. To outline some of the potential model results, two scenarios have been simulated to compare both methods of care regarding overall costs.

**Results:**

The developed simulation model allows comparing health and cost outcomes of different integrated care concepts for the treatment of heart failure patients. Additionally to the simulation of standard outpatient and inpatient care procedures in Austria the approach of a telemedical monitoring system for heart failure patients was implemented in this economic model. With the simulated scenarios it could be shown that under the given simulation parameters the telemedical system can lead to cost savings of up to 8% within the first three years.

**Conclusions:**

The developed model represents a comprehensive tool, which opens a wide field of possible simulation scenarios for the treatment of heart failure patients with special focus on overall cost estimations and reimbursement strategies. The simulated scenarios show that telemedical care has the potential of improved health outcomes and economic benefits.

## Background

The demographic development resulting from the so called “double aging” effect shows that the population distribution has already changed considerably and will further change over the next decades. The proportion of people above the age of 60 increases dramatically. Estimations indicate that in about 20 years every forth will be above 60 and every ninth above 75 years. This expected increase of older people in society poses an immense challenge to the public health care system, since the consequential rise of chronic illnesses represents a not to be underestimated burden [1,2]. About 70% of the population between 70 and 79 years will suffer at least from one of the five most widespread chronic diseases such as heart failure (HF), which numbers among the most internistic illnesses. About 1-2% of people in the United States and in Western Europe are estimated to live with heart failure and prevalence increases because of the aging of population [3-5]. At the age of 45 to 55 years about 1% of the population suffers from heart failure, between 65 and 75 years this value varies from 2 to 5% and rises to about 10% at the age of 80 years and above [6-8]. As a logical consequence expenses for the treatment of these patients will increase noticeably. Health expenditures on chronic heart failure are estimated to account for 1-2% of the total health care budget of western industrialized countries and almost two-thirds of them is due to hospitalizations [9]. Moreover epidemiological data shows that heart failure is the leading cause for hospitalizations regarding patients older than 65 years [6]. It is clear that new strategies for patient management or new methods of medical care such as the possibility of telemedical treatment can be essential for the reduction of the future financial burden. The advantage of telemedical treatment of heart failure patients lies within the earlier detection of symptoms and abnormal vital parameters compared to standard care solutions. Thus treatment at an early stage is possible whereby the health status can be rather stabilized and frequent doctor visits and/or hospitalizations can be reduced and/or prevented [10]. These propositions are supported through the results of many publications and reviews concerning patient outcomes and economic impact of telemedical treatment programs. For example Seto in 2008 performed a systematic review regarding cost comparison between telemonitoring and usual care of heart failure. It was concluded that costs can be reduced between 1.6 to 68.3% by using a telemonitoring system including a component of home physiological measurements. Although telemonitoring systems require an initial investment, they substantially reduce costs in the long term [11]. Klersy et al. in 2011 investigated the economic impact of remote patient monitoring. A cost simulation model was constructed to compare this strategy with usual care. Results show that direct costs for hospitalization are lower for telemonitoring systems with a range from 300 to 1000 Euro per patient. It was concluded that the overall acceptance of remote patient monitoring was encouraged, but there is still a lack of prospectively and uniformly collected economic data [12]. A comprehensive tool such as a model for simulation of different treatment procedures of heart failure patients would provide a lot of possibilities for prospective cost estimations. On this basis the aim of this work is to present a detailed heart failure model with the ability to compare the incurred costs of conventional medical care and different integrated care approaches including telemonitoring of heart failure patients.

## Methods

The model was implemented using the simulation software AnylogicTM^®^ (XJ Technologies, Russian Federation), applying the discrete event modelling technique. This method has a middle to low degree of abstraction and allows the description of event based procedures by the implementation of objects and resources.

The treatment procedures of telemedical and conventional care are not identical, thus two separate versions of the model have been created. Each version contains four modules, whereas the patients are treated in one of the four implemented NYHA classes (New York Heart Association) [13]. Depending on the NYHA class of the patient, the implemented probabilities for decisions regarding different events in the model change. The more severe the heart failure is, the higher the chances for negative events are. As an example NYHA 3 patients are more likely to be hospitalized, have on average a higher length of stay and are more likely to face individual medical procedures (IMP) than NYHA 2 patients.

**Figure 1:**
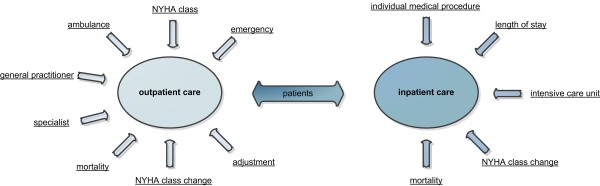
Model overview and influence parameters for the outpatient and inpatient care of heart failure patients.

Additionally every module consists of 2 blocks whereby the first one represents the patient’s treatment outside the hospital (outpatient care) and the second one refers to the stationary treatment process (inpatient care). All differences, based on the use of a telemonitoring system instead of conventional patient care, solely concern the outpatient care. The implementation of the inpatient care process is equal for both versions of the model. Of course different outcomes, such as length of hospital stay or necessary individual medical procedures are modifiable in both model versions. Figure 1 shows a simple overview of the model and outlines the most important influence parameters and resources which were considered to model the outpatient and inpatient care of heart failure patients.

As a main parameter of the general model structure, the NYHA class indicates the severity of heart failure and thus affects for example probabilities for mortalities, admissions to the hospital, intensive care stays and emergency transports. The treatment resources of the outpatient care are the general practitioner, the specialist, the ambulance unit and emergency events. Based on implemented probabilities, these decision items influence the further treatment procedures.

For the inpatient care, patients have different lengths of stay depending on the severity of heart failure and can face admissions to intensive care units and individual medical procedures. The potential improvement or deterioration of the patients’ state of health is reflected by NYHA class changes after the in-hospital stay. As shown in Figure 1, both concepts of care consider implemented morality rates.

Since a model is always an approximation of the real world, the quality of implemented data is important to achieve reliable results. As a data source, the MOBITEL study [14] delivered insights on the effects of the telemedical treatment compared to the conventional approach. Rates of admission (standard and intensive), length of stay, medication and mortality rates could be adopted for the simulated scenarios. Cost estimations for outpatient care resources are based on common fixed rates and for inpatient care on the Austrian DRG system [15]. The model also considers health outcomes of patients undergoing conventional or telemedical treatment. For example the NYHA progression over time, the amount of hospital admissions and mortality rates are integrated in the model.

The validation of the model was performed using the “V-model” development process [16]. In the verification phase it was proven that implementations in each module represent the predefined specifications. The validation process of determining to which degree the functional specifications of the model are fulfilled was based on simulated scenarios with regard to results of the MOBITEL study [14].

### Inpatient care

**Figure 2:**
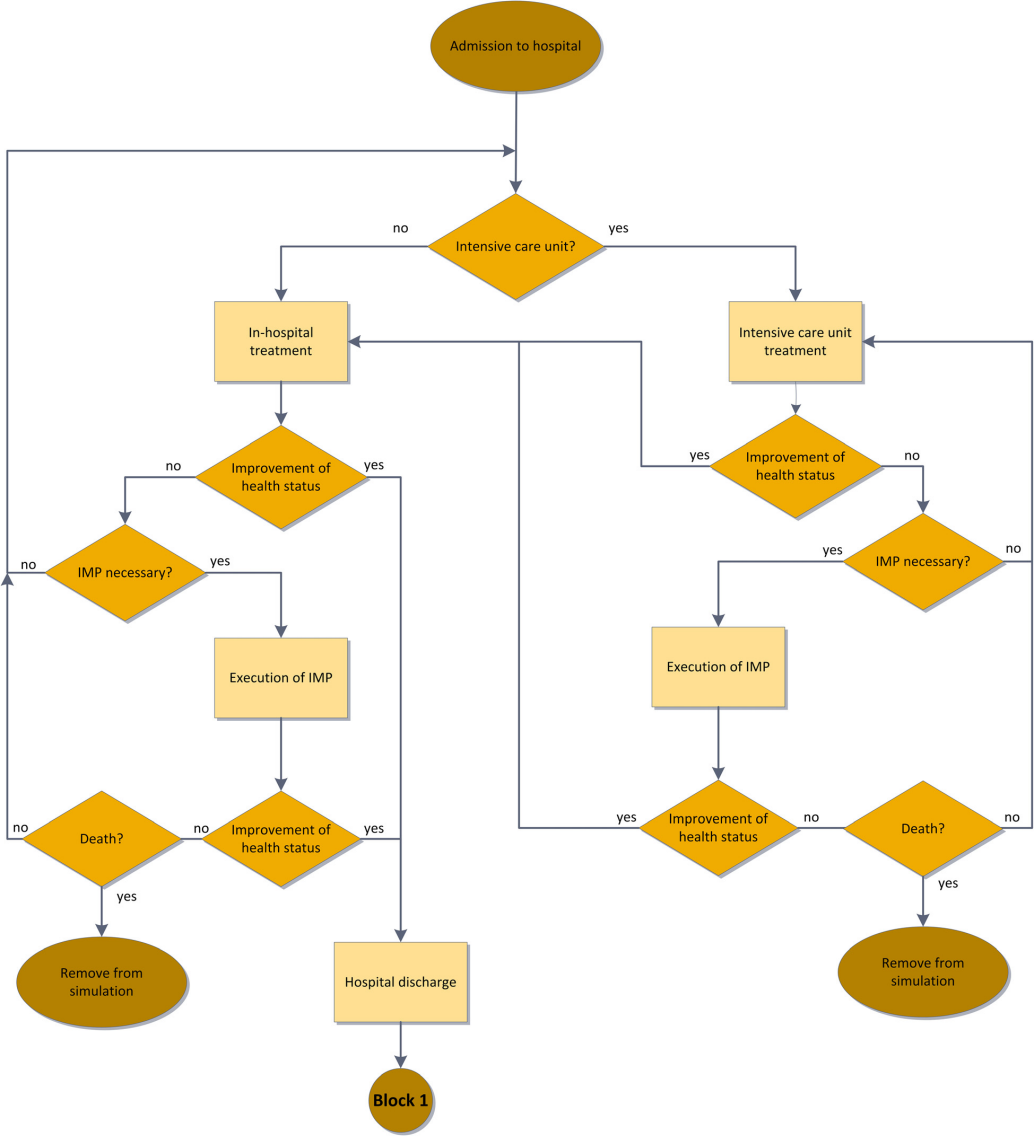
Flowchart of the implemented treatment procedure for inpatient care of heart failure patients.

Principally there are three events implemented in the simulation models which can lead to a hospitalization of the patient: Any emergency, if interventions implicate no improvement of the patient’s health status or if the treating physician recommends the admission to the hospital. For in-hospital treatment the model distinguishes between inpatient and intensive care admissions. Depending on the severity of the patient’s health status, an intensive care treatment can be necessary. The whole procedure of inpatient care is illustrated in Figure 2.

In both, standard and intensive care individual medical procedures based on the Austrian diagnoses related groups (DRG) system can be passed through by individual patients. The expenses of each individual medical procedure are considered in the model. Detailed information on cost estimation is given at the end of the methods section. As you can see in Figure 2 the inpatient care procedures allow the patients to change from standard hospital treatment to intensive care treatment and vice-versa. The patient leaves the hospital if the physical health status improves. This is implemented in the simulation model by the allocation of a specific length of stay in the hospital. After the hospital discharge and a potential change of the NYHA class the patient leaves block 2 and is redirected to block 1, the outpatient care. If no improvement of the patient’s health status takes place, mortality rates are implemented in the inpatient care treatment. Patient death leads to the removal from the simulation.

### Outpatient care – procedure of conventional treatment

**Figure 3:**
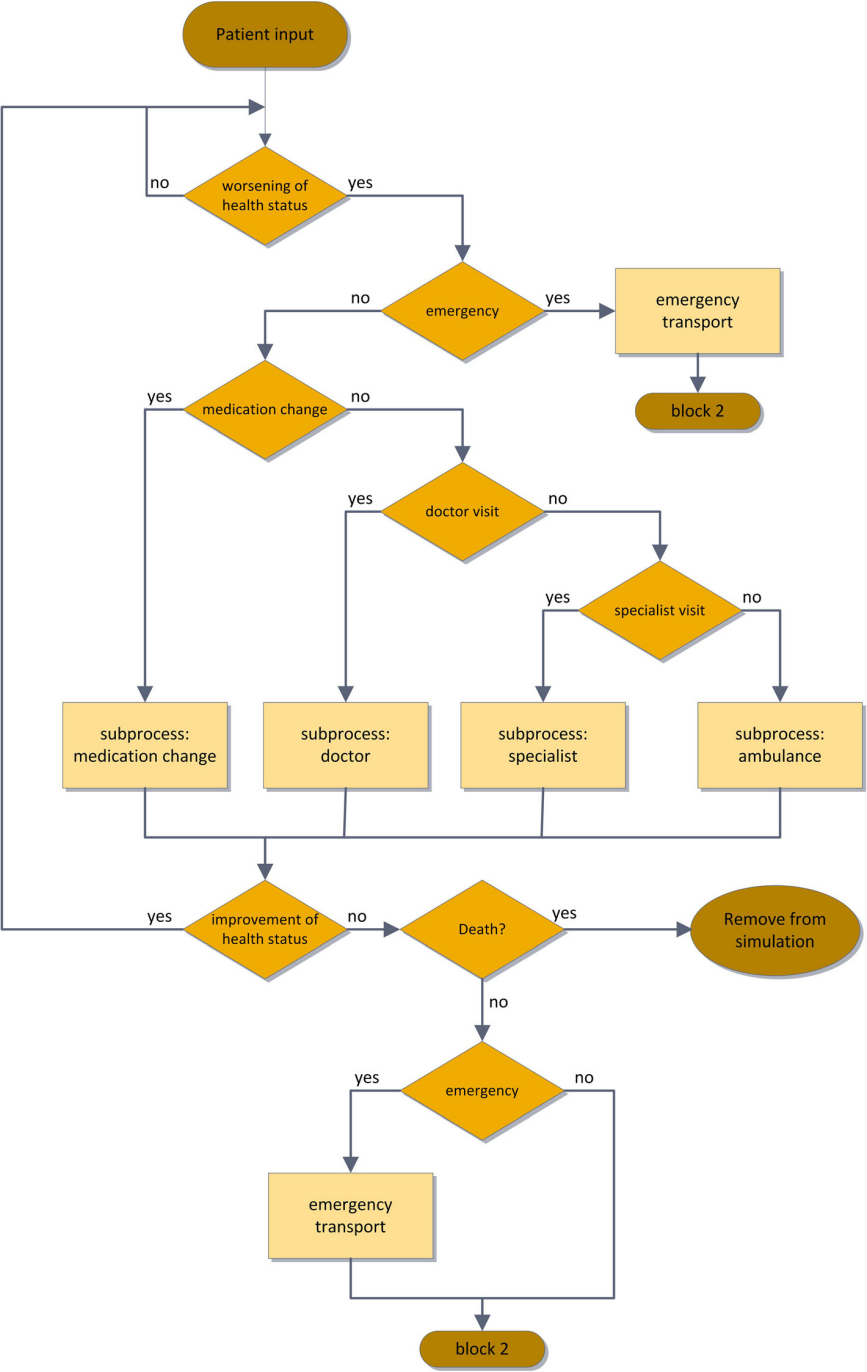
Flowchart of the implemented conventional treatment procedure for outpatient care of heart failure patients.

The outpatient conventional care treatment is illustrated in Figure 3. A worsening of the patient’s health condition, which is reflected by implemented probabilities with regard to the severity of heart failure, is the first decision-step for further treatment procedures. In case of severe decline of health an emergency transport to the hospital is implemented. If no emergency transport to the hospital is necessary there are several possibilities for patient treatment in this simulation block of the model. One sub-process could be the change of medication of the patient. In that case no personal contact to any health care provider is necessary. Alternatively there are three possible health care services: the doctor, the specialist, or the ambulance. If an improvement in the state of health takes place no further events are necessary and the patient is forwarded to the beginning of the simulation block. If no improvement is obtained, patients can die via implemented mortality rates, or the patients get hospitalized via emergency or normal transport, and therefore are transferred to the inpatient care procedure, represented by block 2.

### Outpatient care – procedure of telemedical treatment

**Figure 4:**
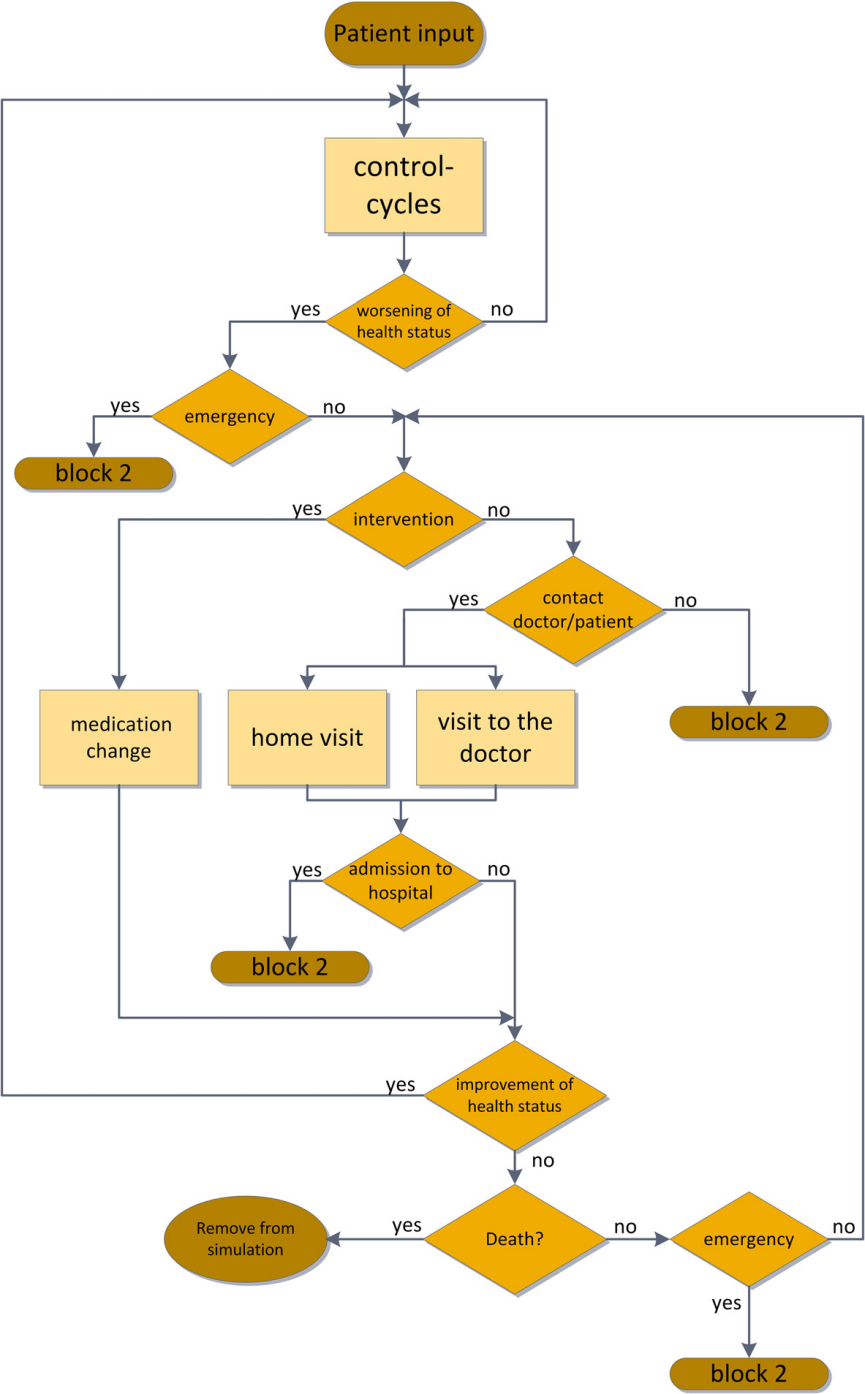
Flowchart of the implemented telemedical treatment procedure for outpatient care of heart failure patients.

The course of the telemedical treatment is based on the most common applications of telemonitoring systems for heart failure patients. The patient is consistently supervised through the telemonitoring system and has to send physiological data such as weight, blood pressure, heart rate and medication once a day to the telemedicine centre [14,17-19]. In case of worsening of the health status the authorized physician is notified. If no emergency occurs, the physician is able to induce appropriate measures. The course of the telemedical treatment can be seen in Figure 4.

In the model the control cycle takes place on a weekly basis. Potentially dangerous changes in the health status of a patient could lead to an event of emergency, which results in a hospitalization of the patient (transfer to the inpatient care, represented by block 2). If vital parameters can be improved, for example via new medication or an adaption of the current treatment, an intervention event is executed in the model. If no intervention is chosen the model offers the possibility of a home visit of the doctor, a visit to the doctor or a hospitalization of the patient. After the face to face contact another decision, whether the treatment can be adapted to improve the patient’s health status or if inpatient care is unavoidable, is implemented in the simulation model. Finally, if no improvement of the patient’s physiological parameters can be achieved, hospitalization is possible or mortality rates are implemented in the model to consider possible death scenarios. After this process the patient can undergo a change in his NYHA class, depending on improvement or worsening of his health status. In both cases, the conventional and the telemedical care, extramural mortalities have been implemented separately for each NYHA class.

### Costs

**Figure 5:**
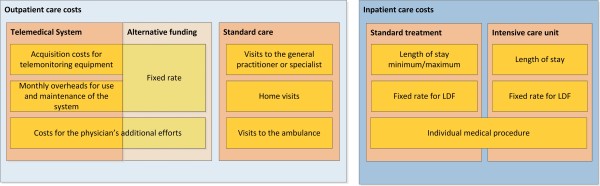
Illustration of the composition of the overall costs divided into inpatient and the outpatient care costs including alternative financing.

For economic analyses of the course of treatment of each patient, outpatient as well as inpatient care costs are considered in the simulation model. Since a decrease in hospitalizations is expected when patients are equipped with a telemonitoring system and the inpatient care accounts for the vast majority of the total costs for heart failure treatment, it is very important to implement these expense factors as properly as possible. Therefore the calculation in this model is based on the Austrian diagnosis-related-groups (DRG) system. In the DRG model, hospital stays are grouped into procedure-oriented diagnosis-related case flat rates (LDF is the German abbreviation of “Leistungsorientierte Diagnosenfallgruppe”, which can be translated as “procedure-oriented diagnosis-related case group”). Every case flat rate has a typical length of stay (defined by a concrete minimum and maximum) allocated to it, during which a specific point score is reimbursed to the hospital. For the diagnosis of heart failure the minimum and maximum are currently defined as 4 and 12 days. If the length of stay exceeds beyond the maximum, supplementary points are added to the case flat rate for each additional day. In contrast, if a hospital stay is shorter than the minimum length of stay, a reduced flat rate is reimbursed [15]. Specific categories of diseases and degrees of severity are taken into account in different LDF-groups. Therefore LDF points and length of stay in the simulation model can be varied for each NYHA group. In addition to these costs resulting from standard hospital treatment expenses from intensive care units are considered in the model. Stays at intensive care units are reimbursed by a daily supplementary score in addition to the normal case flat rate and can be regarded as an indicator of severity for heart failure. Following this the implemented LDF rate and the length of stay at intensive care units are variable for each NYHA group and result in an additional cost contribution of inpatient care. If an individual medical procedure (e.g. expensive surgery) is necessary, a procedure component will be considered in the model and additionally increases the total expenses of inpatient care. An illustration of the overall cost composition including inpatient and the outpatient care costs is shown in Figure 5.

Outpatient care costs are divided into costs for the standard care process and expenses for the telemedical system, if applicable. Standard care costs comprise expenditures of visits to the general practitioner or a specialist, home visits or a medical examination in an ambulance.

For the telemedical treatment of patients different adaptable variants of financing the telemedical system are implemented in the model. One opportunity is to consider a fixed monthly rate per patient, including a lending fee for the equipment and costs for the maintenance of the system. Another possibility is to split up equipment costs and costs for the service of the system. In this case a separate amount for the acquisition of the telemonitoring equipment will be considered, as well as monthly overheads for the general use of the system and maintenance. In both financing concepts additional expenses for the physician’s efforts of monitoring the patients are considered in the model. All of the described parameters can be individually entered for various simulations and summarized to the total costs of outpatient care.

## Results

The result of this work is the development of a stochastic simulation model, which allows to compare health and cost outcomes of different integrated care concepts for the treatment of heart failure patients. Additionally to the simulation of standard outpatient and inpatient care procedures in Austria the approach of a telemedical monitoring system for heart failure patients was implemented in this economic model. Model parameters can be directly modified via input masks on the simulation screen to set, for example, mortality rates, hospitalizations, costs for visits to the ambulance, amount and type of individual medical procedures and class changes to conduct specific simulation runs. In total 3 input masks allow the adjustment of the event-probabilities, which then influence the patient’s treatment procedure, but do not interfere with the general model structure. As a first step for each simulation the amount and the distribution of patients under investigation can be adjusted for each NYHA class. In a second step the probabilities for events occurring in the outpatient and inpatient care can be specified. This refers to the parameters previously described in the methods section, for example the distribution of home visits, doctor and specialist visits, in-hospital length of stay, mortality, intensive care unit treatment and so on. Finally the model allows the separate adjustment of expenses for standard care events, telemedical procedure and hospitalization as described above (see Figure 5). In addition the amount of Euros per procedure-oriented diagnosis-related case group point is adjustable for possible future adaptations.

The time horizon for running the simulation is variable and can be chosen by the user. One time-step in the model environment is equivalent to one day in the real world. With the opportunity to simulate patient treatment over a longer time span as in most of the conducted studies (follow-up between 6 and 12 months) it is possible to determine the breakeven point for different concepts of care. This point changes noticeably by twisting one of the many screws given in the model such as the hospitalization rate, the length of stay, the overall NYHA distribution of patients, telemedical treatment costs or the probabilities for NYHA class change.

Each simulation produces a set of graphs and tables describing the incurrence and constitution of the overall treatment expenses for the virtual heart failure patients. Additionally the development of the patient collective for each NYHA class and its course through the model structure can be observed over time. Moreover the developed model offers the chance to estimate the most important influence parameters and their impacts on different treatment processes by using sensitivity analysis.

To outline some of the potential model results, a comprehensive scenario in regard to overall outcomes of telemedical and conventional care has been simulated. Intramural NYHA class changes, in-hospital mortality rates as well as different concepts for the calculation of costs for the telemedical system have been used.

Tables 1 and 2 show a summary of input parameters and treatment costs for the simulated scenarios. Visits to the ambulance, costs of medication, costs for transport and extramural mortalities have not been included in these scenarios.

**Table 1:** summary of the input parameters for the simulated scenarios

**Parameter**	**Conventional**	**Telemedical**
	**care**	**care**
Median length of stay	10 days [17]	6.5 days [17]
Median length of stay	2 days	2 days
(Intensive unit)		
Hospitalizations	32.08%^a^[17]	20.37%^a^[17]
Visits to general practitioner	1.42^b^[20]	2.84^b^[20]
Visits to specialist	0.3^b^[20]	0.46^b^[20]
Intensive care treatment	12.6%^c^	12.6%^c^
In-hospital mortality	7.1%^c^	7.1%^c^

^a^… probability for the time period of half a year.

^b^… amount of visits for half a year.

^c^… probability per stay.

**Table 2:** cost parameters for the inpatient and outpatient care

**Health care resource**	**Costs**
Visit to the general practitioner	25 €
Visit to the specialist	31 €
In-hospital stay	1523 €[15]
Intensive care stay	2496 €[15]

The simulations are based on a starting pool of 100 patients over the time span of three years, with the following NYHA distribution: 0% NYHA 1, 61% NYHA 2, 37% NYHA 3 and 2% NYHA4 [8]. The final outcomes are obtained by taking the mean of 10 individual simulation runs to reduce randomness of the stochastic model. On one side, the expenses for the telemedical system were implemented as a monthly fixed rate of 100 € per patient (TM_scenA) and on the other one acquisition costs for the telemonitoring equipment were taken into account with 1000 € for each system, monthly overheads for the use and maintenance of the system with 500 € and costs for the physician’s additional efforts with 40 € for each patient per month (TM_scenB).

**Figure 6:**
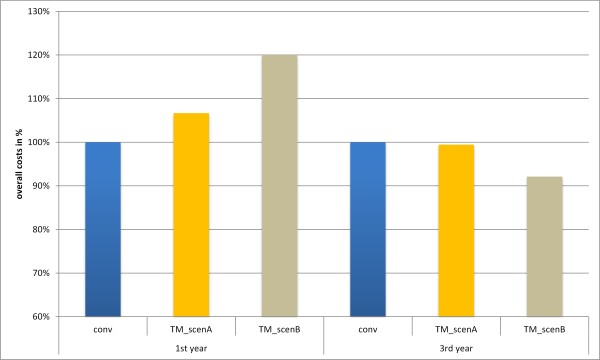
Bar diagram of the overall costs for the simulation of conventional and telemedical care over a time span of 3 years.

In Figure 6 the results of the overall costs are compared. The telemedical scenarios A and B are matched with the conventional care process, which was set to 100%. The telemedical treatment is not cost efficient after the first year. Depending on the method of funding for the telemedical system the overall costs exceed the expenses for the conventional care up to 20%. Under the given simulation parameters the break-even point for both telemedical calculations lies approximately at two years and cost savings of up to 8% can be achieved within the first three years by using a telemedical treatment approach. Regarding health outcomes it could be assessed that 14 patients treated via conventional care died during the time period of three years in comparison to seven who received telemedical treatment.

## Discussion

### Simulation model

There are different decision-analytic modelling approaches to estimate the cost effectiveness of health technologies for chronic heart failure described in the literature. Goehler et al. identified 34 modelling studies investigating different interventions [21]. Most of them used Markov models (27) and a few used mathematical equation sets (4) or discrete event simulation (3) models. In principle there is no general recipe which modelling approach should be chosen, however discrete event simulation models (DES) have some essential advantages [22-24]. One of these characteristics of DES models is the ability to express the experience of individuals over time in terms of events and their consequences rather than reflecting the world as a series of states used in Markov simulations [23]. It seems to be well understandable to model the reality in terms of events that can happen over the course of health care procedures. DES methods are more efficient in using attributes, in our case the NYHA classification of the virtual patients. On the one hand changes of the severity of the disease can be considered and thus influence following treatment procedures and on the other hand tracking the patients enables the option to assess the distribution of NYHA classes at follow-up time, not only as usual at baseline. Relating to the target group of HF patients, using NYHA classification as an assessment tool for disease progression is a common approach in decision analytic models. Apart from the modelling approach previously published models mainly investigate pharmacological treatment versus standard therapy of heart failure patients. For example Caro et al. developed discrete event simulations to compare the course of HF patients after the implantation of an ICD (implantable cardioverter defibrillator) with additional amiodarone medication [25,26]. A linear model based on an Excel platform from Klersy et al. investigates the economic impact of patient remote monitoring compared to usual care. The model is very simply structured and only considers costs for hospitalizations [12]. However, so far no comprehensive decision analytic model was found in literature, which was developed to compare the incurred costs of conventional medical care and different integrated care approaches (outpatient and inpatient courses) including a telemedical monitoring system of heart failure patients. This is in marked contrast to the high number of different studies regarding economy impact, cost predictions, or cost-effectiveness on these possible treatment concepts. Müller et al. in 2010 summarized experiences from different telemedical support projects in Germany. It was concluded that chronic heart failure patients benefit from telemedical monitoring with transfer of vital parameters [18]. But it is still an open question which patient with regard to NYHA classification benefits most from the telemedical care system. For future cost savings and/or reimbursement strategies this will be one of the most important questions which have to be clarified. First experiences with simulation models showed that the cost effectiveness of telemedical treatment strongly depends on the collective of patients (amount, NYHA class) [27,28]. Therefore an extension of the simulation model by using attributes for NYHA classification and tracking the virtual patients was unavoidable [29].

### Cost estimation

For the first time a comprehensive simulation model regarding cost estimations for HF patient treatment, including outpatient as well as inpatient care was developed. Compared to other existing models which primarily consider hospitalization cost factors, the total cost estimation is implemented in a more exact approach. Additionally the developed model takes costs of outpatient care, costs for monitoring the patients and equipment costs into account. In other studies regarding economic analyses of HF telemonitoring systems these items were considered separately, however not combined in one comprehensive work. In his review Seto concluded that most of the studies included hospitalization costs and telemonitoring equipment costs, but only one study discussed the direct impact of HF telemonitoring on expenses for the patient [11]. A consideration of the so called indirect costs for the patients, currently not implemented, seems to be an improvement of the presented model. For example travel costs for visits to physician or to ambulatory as well as reductions in income play an important role for each individual. Additionally the decrease in number of home vistits seems to be a potential area of cost savings. The modular concept of the developed model enables the consideration of indirect costs. Further the model structure allows the separate analysis of all implemented cost contributions. Medication costs have not been considered so far and should be implemented in the next extension step of the simulation model. However, increasing model complexity will negatively influence the traceability and comprehension of the existing model. Therefore a balance between exact reproductions of real processes and their cost contributions and traceability of the model has to be kept in mind for future extensions.

Compared to existing models and estimations for cost savings regarding telemedical care, the often overlooked implementation costs for the telemonitoring system have been considered in the developed model. In addition different adaptable variants of financing the telemedical system are taken into account. Regional disparities (age distribution, population, etc.) are another deciding factor, which can account for the difference between potential success or failure of telemedical systems. Depending on the quality and existence of data the model can be used to map the present method of care in for example a city or a state and to compare it with alternative treatment processes for heart failure patients.

Considering all contributions the model allows the simulation of various concepts of reimbursement modalities, such as tariffs for ambulatory patient control, per capita monthly rates for a service contract or device based reimbursement. The systematic search from Seto also found articles, where co-payments by the patients would be accepted to access telemedicine service instead of travelling to the physician [11]. Simulation results received from such different concepts can support third-party payers when defining reimbursement strategies. Another application for the developed model is budget impact analyses, where hypothetical cohorts of patients can be observed over a defined simulation time. The economic analyses from Klersy et al. pointed out that the limited follow-up time of most existing studies [12] has an important influence. As mentioned above the time horizon for running simulations is variable, accordingly the follow-up time can be extended by using the developed simulation model.

The results of the simulated scenarios illustrate the financial outcomes for both methods of care, with different approaches to the calculation of the overall costs for the telemedical system. It can be seen that under the given simulation conditions the telemedical care has a potential for cost savings after the first two years, which increases noticeably depending on the variant of funding. The choice of the distribution of NYHA patients strongly affects the overall costs for both methods of care. With further simulations and analyses of the model, different results can be discussed, whereby the effectiveness of the telemedical treatment of heart failure patients is in the centre of attention. Under the given simulation parameters a reduction of deaths could be achieved, however it has to be mentioned that this is based on in-hospital mortality only.

With the developed model different scenarios can be simulated and analysed to develop sustainable health care solutions. As previously described the model is based on many different probabilities which strongly influence the simulation results. Therefore it has to be considered that NYHA-related data is specific to the target study group and the choice of data used for the model should be made carefully. Depending on the quality of implemented data (e.g. NYHA related transition probabilities, mortality rates, etc.) valuable predictions for future developments can be achieved, representing real life scenarios with good precision.

## Conclusions

The developed model represents a comprehensive tool, which opens up a wide field of possible simulation scenarios for the treatment of heart failure patients with special focus on overall cost estimations and reimbursement strategies. The simulated scenarios show that telemedical care has the potential of cost savings. However, it has to be stated that these are preliminary results. With the amount of adjustable parameters it is possible to simulate a variety of scenarios to clarify under which condition a telemedical provision for heart failure patients leads to improved health outcomes and economic benefits. Based on the developed model, prospective planned cost reduction activities, such as the possible implementation of telemedical care can be assessed and used for decision making in health care systems.

## Abbreviations

HF: Heart failure; NYHA: New York heart association; DRG: Diagnoses related groups; LDF: Leistungsorientierte Diagnosefallgruppe (can be translated as procedure-oriented diagnosis-related case group); DES: Discrete event simulation; ICD: Implantable cardioverter defibrillator.

## Competing interests

The authors declare that they have no competing interests.

## Authors’ contributions

JS drafted the structure and overall content of the paper. AL was responsible for the model and the scenarios. Together both authors finalized, read and approved the final manuscript.
